# A State-of-the-Practice Review on the Challenges of Asphalt Binder and a Roadmap Towards Sustainable Alternatives—A Call to Action

**DOI:** 10.3390/ma18102312

**Published:** 2025-05-15

**Authors:** Swathi Malluru, Sk Md Imdadul Islam, Ahmed Saidi, Anil Kumar Baditha, Gordon Chiu, Yusuf Mehta

**Affiliations:** 1 Center for Research and Education in Advanced Transportation Engineering Systems (CREATES), Rowan University, Glassboro, NJ 08028, USA; mallur54@rowan.edu (S.M.); islams62@students.rowan.edu (S.M.I.I.); ahmeds@rowan.edu (A.S.); baditha@rowan.edu (A.K.B.); 2 Phenegra Corporation, Summit, NJ 07901, USA; dr.chiu@phenegra.com

**Keywords:** challenges, asphalt binder, performance, environmental impact, modifiers, alternatives, sustainability

## Abstract

Increasing traffic loads, extreme climatic conditions, and environmental regulations highlight the need to re-evaluate the use of existing asphalt binders in pavement construction. This paper examines the limitations of conventional and modified asphalt binders by incorporating a comprehensive literature review that focuses on performance, environmental impact, and economic issues. Studies show that binder grade selection, mixing and compaction temperatures, and ageing affect pavement performance and may reduce pavement service life by 10% to 30%. Although modifiers such as polymers and nanomaterials can improve rutting and moisture damage resistance by up to 50%, they have limited effects on fatigue and thermal cracking resistance. Moreover, these modifiers can affect the asphalt mixture production process due to source variability, leading to complex mixing methods, increased cost, and higher emissions. Additionally, high-temperature asphalt mixture production increases air pollution by 250%, causing health risks. Furthermore, asphalt binder and mixture production account for over 50% of the total pavement costs, and the rising asphalt binder prices place a burden on highway budgets. This review highlights the critical research gaps including source variability, testing and mixing methods, and environmental impact of modifiers and provides a future roadmap for developing cost-effective and sustainable alternatives and their practical implementation.

## 1. Introduction

Asphalt binder is a complex organic compound and a viscous liquid composed of hydrocarbons obtained through the non-destructive distillation of crude oil during the petroleum refining process and is a commonly used material in road construction because of its unique properties such as strength, durability, flexibility, and cost-effectiveness, which have made it a long-preferred choice for building roadways [1,2,3]. Asphalt from the distillation process possesses higher plasticity, adhesion, and hydrophobicity than that from the air-blowing technique, indicating that production technology influences asphalt properties. Additional factors such as oxidation, evaporation of lower molecular weight components, and physical hardening of components cause structural changes in asphalt binder [4]. Asphaltenes and resins are the primary components in asphalt binder that contribute to rheological properties and temperature susceptibility, which directly influences the binder’s suitability for various applications [5,6].

The practice of mixing hot asphalt binder with aggregates to form paving material has been in use for over 100 years and has consistently proved to be an effective and durable material for asphalt pavement construction worldwide [7]. Asphalt binder plays a major role in the performance, environmental, and economic issues associated with the asphalt pavement life cycle. Distresses such as fatigue cracking, rutting, thermal cracking, and moisture damage, which are primarily driven by the asphalt binder, occur during the operation and service stages of asphalt pavements caused by traffic and climatic conditions such as temperature fluctuations, extreme heat, and freeze–thaw cycles. These distresses ultimately contribute to a decline in pavement performance and service life [8].

The need to extend the service life and improve the performance of asphalt pavement led to the development of polymer-modified asphalt binders. These binders are developed to address the challenges of conventional binders, specifically in terms of performance and durability. The commonly used polymers for binder modification include polyethylene (PE), polypropylene (PP), polystyrene (PS), polyethylene terephthalate (PET), ethylene–vinyl acetate (EVA), ethylene–butyl acrylate (EBA), styrene–butadiene–styrene (SBS), styrene–isoprene–styrene (SIS), styrene-butadiene rubber (SBR), and styrene–ethylene/butylene–styrene (SEBS). These polymer modifiers have different effects on asphalt properties. For instance, SBS, SBR, and EVA reduce the temperature susceptibility of asphalt, increase its cohesion, and improve its rheological properties, while crumb rubber increases asphalt’s viscosity and improves its resistance to both rutting and low-temperature cracking [9,10,11,12,13]. Moreover, the use of nanomaterials, waste plastic, and waste cooking oil (WCO) has been shown to improve the mechanical and rheological properties of the asphalt binder [14,15,16].

Building on the need to enhance asphalt binder performance through its modification, many nations, including the United States of America (USA), China, and others, have investigated the incorporation of various additives. Although asphalt modifiers offer notable performance improvements for asphalt pavements, several studies reported that their application is associated with certain limitations, including high costs, storage stability concerns, the need of standardized mixing procedures and specifications, and environmental impacts related to production and disposal [15,17,18,19,20,21,22,23,24,25,26]. Researchers also found that, in addition to these limitations, other factors have also contributed to pavement deterioration to some extent, such as climate change along with the emerging traffic load patterns with the growing presence of electric vehicles [27,28,29,30]. The combined effect of climate change and changing traffic patterns adds new complexity to pavement performance. While existing literature addresses the limitations of conventional and modified asphalt binders in isolation, there is a critical research gap in systematically synthesizing these challenges into a holistic framework that accounts for their interdependencies. These limitations also underscore the urgency of a comprehensive evaluation of sustainable alternatives capable of supporting the development of eco-friendly and resilient asphalt pavements. This study aims to provide a comprehensive overview of the challenges associated with the asphalt binders currently in use. Furthermore, it explores alternatives binders that offer cost-effective, environmentally friendly, and performance-enhancing solutions. Additionally, the study examines the limitations of these alternative binders and identifies critical gaps, underscoring the need for rapid development and adoption of sustainable binder alternatives in the near future to mitigate the above-mentioned challenges.

## 2. Goals and Objectives

The goal of the study is to identify the challenges of asphalt binder including modifiers, highlight research gaps, and underscore the need for more sustainable alternatives. The objectives to achieve the goal are as follows:

- Examine the challenges associated with asphalt binders.
- Identify alternative binders or technologies that aim to mitigate these challenges of conventional binders while promoting sustainability, performance, and cost effectiveness.
- Evaluate the limitations of these alternative solutions and highlight the research gaps for further development of sustainable alternatives.

## 3. Methodology

**Figure 1:**
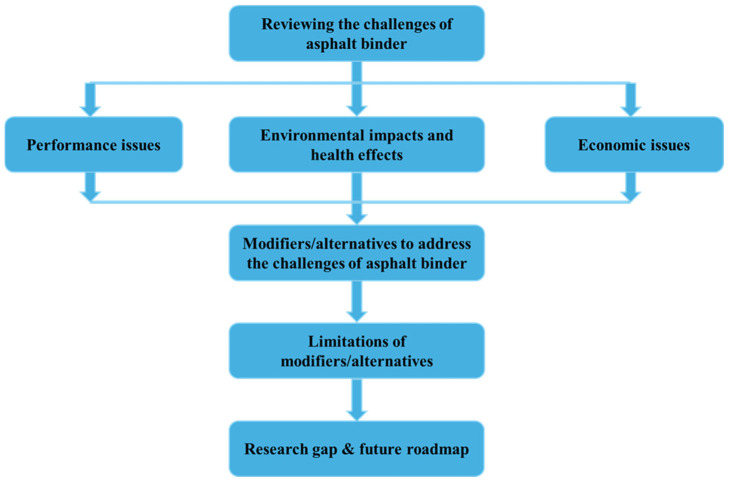
Research approach of the study.

A systematic approach was developed to review the challenges associated with asphalt binders in terms of performance, environmental impact, and economic aspects. After a comprehensive review, potential modifiers or alternatives that could mitigate these challenges are identified. Finally, the limitations of these modifiers/alternatives are examined to highlight the research gap. Figure 1 illustrates the research approach of the study.

### Bibliometric Approach

A bibliometric analysis was conducted to systematically map research trends, key contributors, and knowledge gaps in challenges facing conventional and modified asphalt binders. Data were extracted from Scopus, Web of Science, and Google Scholar considering conference, book chapter, and journal publications from the 1990s to most recent ones in 2025 using keywords such as “asphalt binder”, “polymer-modified asphalt”, “nanomaterial-enhanced asphalt”, “effect of rejuvenator in asphalt binder”, “polymer modification costs”, “environmental sustainability with asphalt binder modification”, “nanomaterial dispersion”, “rejuvenator efficiency”, and “economic feasibility and environmental impact of asphalt binder”. Inclusion criteria prioritized peer-reviewed studies addressing pavement performance, cost, and sustainability. The study presented a transition from early conventional binders to recent advancements in polymer-centric research, nanomaterials, and recycling agents with significant contributions to the asphalt industry from researchers in the USA, China, India, Middle Eastern countries, and Europe. Conventional binders were linked to performance issues and environmental compromises, whereas modified binders struggled with elevated production costs, phase separation, potential toxicity, and variable effectiveness. The analysis underscored the need for studies on effective combinations of alternative binders and modifiers and on their long-term performance and environmental evaluations, proposing future directions emphasizing eco-friendly materials.

## 4. Challenges of Asphalt Binders

The challenges of conventional asphalt binders in terms of performance, environmental impact, and economic aspects are significant. Performance concerns include cracking and rutting, while environmental issues arise from emissions and resource consumption. Economically, fluctuating material costs and long-term maintenance expenses add complexity. These aspects are explored in detail in the following sections, highlighting potential solutions and advancements.

### 4.1. Factors Affecting the Long-Term Performance of Asphalt Pavements

#### 4.1.1. Material Selection

The quality of an asphalt binder depends on its source, as its properties vary significantly from one crude source to another, which will change the overall characteristics of the asphalt mixture [7]. Selection of grade of asphalt binder appropriate to prevailing climatic conditions plays a crucial role on the durability of asphalt mixtures as it is affected by the temperature fluctuations. Inappropriate asphalt grade causes premature fatigue cracking, requiring early maintenance [31]. At high temperatures, asphalt softens and undergoes permanent deformation under traffic loading, and at intermediate or low temperatures, asphalt becomes stiff and brittle and is prone to cracking due to load or freeze–thaw cycles [32,33]. A low-viscosity-grade asphalt can induce rutting at 60 °C in asphalt mixtures with high asphalt content, and a high viscosity will cause thermal cracking at low service temperatures [34]. The severity of low-temperature cracking is a function of temperature and the stiffness of the asphalt binder type [35].

#### 4.1.2. Construction Practices

The type of asphalt binder impacts the mixing and compaction temperatures of asphalt mixtures. Binder with lower viscosity requires low mixing temperatures compared to higher viscosity binders for effective coating of the aggregates. The high mixing temperatures of stiffer binders will lead to premature aging, affecting the durability of asphalt pavements. In general, a decrease in the compaction temperature of the asphalt mixture increases the stiffness of the asphalt, resulting in insufficient compaction, affecting the air voids in the mixture. In most cases, stiffer binders require higher compaction temperatures and effort, making it difficult to achieve the required level of air voids [34,36,37,38]. One of the studies conducted by Linden R.N et al. (1989) [39] examined the relationship between asphalt mixtures’ density and performance, and they stated that a 1% increase or decrease in air voids over the base air void level of 7% can improve or reduce the pavement’s service life, respectively. Furthermore, several laboratory and field studies show that a 1% reduction in air voids below the base air void level improved the fatigue performance by 8.2% to 43.8% and rutting resistance by 7.3% to 66.3% [40].

#### 4.1.3. Service Life

After construction, asphalt pavements are subjected to traffic load and varying climatic conditions that play a crucial role in their deterioration. Environmental factors such as temperature, moisture, sunlight, ultraviolet (UV) light, and traffic load affect the material properties and long-term performance of asphalt pavements. The adverse effects of the environment and traffic load are outlined in the following sections.

##### Asphalt Ageing

Environmental factors such as temperature and rainfall have detrimental effects on the durability of asphalt mixtures. One of the critical factors for the damage is the ageing of the asphalt binder due to environmental conditions [41]. Ageing is oxidation and loss of volatile compounds from the binder due to long-term exposure to sunlight, UV radiation, and extreme weather conditions [42]. Ageing results in increased viscosity and stiffness, reduced elasticity of asphalt binder, reduced cracking resistance and mechanical performance, and poor macro-structure continuity in asphalt mixtures [43]. Ageing is classified into two categories: short-term and long-term. Short-term ageing occurs during the construction phase, while long-term ageing occurs throughout the service life [44]. Short-term ageing is mainly affected by the high temperature of the asphalt mixture and occurs during the mixture production, transportation, and paving processes. Oxidative degradation, which is the loss of volatiles during short-term aging, has a significant impact on asphalt properties due to increased temperature and air exposure. This, in turn, results in a greater susceptibility of asphalt mixtures to cracking [45]. Long-term ageing is significantly affected by UV radiation and the rising environmental changes, and global warming further increases the intensity of UV radiation, making it a serious concern. UV radiation can change the chemical composition and physical and rheological properties of asphalt binder and accelerates the aging of asphalt [2]. Different wavelengths of UV light induce varying ageing effects on asphalt binder, and the wavelength of UV radiation, when matched with the binder polymer’s sensitivity wavelength, will result in degradation of the asphalt binder. The depth of UV ageing in the asphalt binder can exceed 2 mm after the 15-day ageing period, showing the downward diffusion of aged molecules into the fresh asphalt in the lower layers of the binder [46]. This indicates that UV ageing progresses gradually from the surface to the lower layers as UV ageing time increases. Additionally, prolonged exposure to UV radiation induces shrinkage stress in the asphalt binder surface, leading to surface cracking [47]. Therefore, these challenges necessitate the development of anti-UV ageing additives with UV absorption and UV blockers that can improve the anti-UV ageing performance of asphalt binder and mixtures.

##### Effect of Temperature and Traffic Load

Asphalt pavements develop various distresses due to the combined effect of temperature and traffic load that leads to the pavement’s failure [48]. Fatigue cracking is one of the major distresses and causes of failure in asphalt pavements across many countries [49]. Thermal or low-temperature cracking in asphalt pavements occurs due to the combined effect of tensile stress caused by significant reduction in temperature and traffic load [50,51]. In cold regions, thermal cracking contributes to about 37.5% of all the distresses [52]. The repeated traffic load during the use phase of an asphalt pavement causes a permanent depression in the wheel path at elevated temperatures, leading to rutting [53]. Rainfall is the other environmental factor after temperature that significantly affects the durability of asphalt pavements [54]. The intensity of rainfall significantly affects moisture damage. A study by Neupane and Wu (2025) [55] reported that heavy rainfall with intensity above 7.6 mm per hour accelerates moisture-induced damage and weakens the pavement integrity. Moisture-induced damage, caused by the stripping of asphalt binder from the aggregate’s surface, leads to progressive degradation in the form of multiple distresses such as raveling, potholes, rutting, and cracking, affecting the durability and life-cycle cost of the pavement [56,57]. Dong et al. (2023) [58] found that the fatigue cracking resistance and low-temperature performance of high-penetration-grade binder is 200% and 80% more when compared to the low-penetration-grade binder, respectively, while the rutting resistance and moisture damage resistance of low-penetration-grade binder is 150% and 110% higher than the high-penetration-grade binder. According to the study of Radhakrishnan et al. (2018) [59], the higher-viscosity-grade binder can improve the rutting resistance by approximately 400% compared to a lower-viscosity-grade binder. A study by Ji et al. (2023) [60] found that the rutting of high-penetration-grade binder is 80% higher while the fatigue resistance is 30% lower than a low-penetration-grade binder. Additionally, the high-penetration-grade binder demonstrated better moisture damage resistance than the lower-grade-binder.

##### Effect of Extreme Climate Events

Asphalt pavements contribute to climate change through various phases of their life cycle; on the other hand, climate change also significantly impacts the pavement performance. Temperature and precipitation are the two major climatic factors contributing to pavement deterioration. The service life of asphalt pavement can be reduced by 20% due to climate change [61,62,63]. An increase in temperature can increase the rutting by 9% to 40% and fatigue cracking by 2% to 9% [64].

### 4.2. Environmental Burdens and Health Effects of Asphalt

Asphalt binder and mixture production, along with construction activities, emit carbon and other harmful gases into the atmosphere, significantly impacting the environment and construction workers. The environmental impact in terms of carbon emissions and urban heat island (UHI) are discussed in the sections below.

#### 4.2.1. Environmental Burdens

Global warming has been the most debated topic in recent times due to its harmful effects on both ecosystems and human health. The transportation sector has been the major contributor for carbon emissions for the past century, accounting for the largest portion of about 28% among other economic sectors [65]. Asphalt pavements are the main sources of greenhouse gas (GHG) emissions in the transportation sector [63]. Construction and maintenance activities consume fossil fuels and emit CO_2_ and other harmful gases that adversely affect the environment and human health [66]. The environmental burdens of asphalt binder and asphalt pavements in terms of carbon emissions, UHI, and health effects on construction workers are explained in the following sections.

##### Carbon Emissions

Asphalt binder and mixture production are major contributors to air pollution within the pavement lifecycle stages, especially in regions with high temperatures and abundant sunlight. Hazardous organic and particulate matter generated from asphalt pavements causes more serious pollution than the conventional internal combustion engine (ICE) vehicles [67]. Carbon monoxide (CO), nitrogen dioxide (NO_2_), sulfur dioxide (SO_2_), ground-level ozone (O_3_), and particulate matter (PM2.5 of width 2.5 µm and PM10 of width 10 µm) are identified as the major pollutants by World Health Organization (WHO) to protect public health and the environment [68]. An increase in the asphalt surface temperature between 40 °C and 60 °C can double pollutant emissions, which is common in regions like the European Baltic Sea, California, and areas near the equator [69]. A 12 °C decrease in the temperature of a high-temperature mixture reduces the emissions by 25%, while for low-temperature mixtures, the reduction is 50%. A study by Chlebnikovas et al. (2023) [70] found that paving asphalt mixtures increases air pollution by 250% compared to the normal condition.

The life cycle assessment (LCA) method is widely used to assess the environmental impact of asphalt pavements as it provides theoretical support for reducing energy consumption and emissions [71,72,73,74]. Using the LCA method, the source and magnitude of GHG emissions of asphalt pavement can be estimated. The life cycle of asphalt pavement involves five phases: material production and transportation, construction, maintenance, use, and end-of-life. The emissions due to CO_2_, CH_4_, and N_2_O are grouped under the global warming impact category. SO_2_, NO_2_, CO, hydrocarbons (HC), non-methane volatile organic compounds (NMVOC), and heavy metals such as cadmium (Cd), arsenic (As), lead (Pb), and mercury (Hg) are considered as inventory loadings for human and ecotoxicity [75]. The material phase involves the production of asphalt binder, aggregates, and asphalt mixture. Table 1 presents the impact results from a life cycle inventory of asphalt binder and asphalt mixture production and material phases for five impact categories, with asphalt binder production being the major contributor to emissions. Global warming during the material phase is due to asphalt binder and mixture production. Asphalt production is solely accountable for human and ecotoxicity during the material phase. The mixture production phase accounts for 54%, while materials production contributes 43% of the total GHG emissions during asphalt pavement construction [66]. Additionally, emissions from asphalt binder and mixture production have greater than 50% impact on climate change and depletion of fossil fuels [76]. LCA studies from different countries (Table 2) revealed that the material phase of the asphalt pavement life cycle has significant environmental impact [77,78,79,80,81,82].

**Figure 2:**
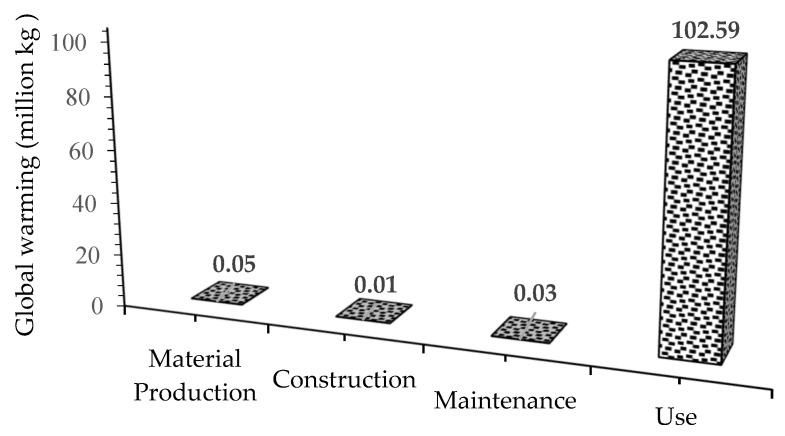
Global warming contribution of each phase of asphalt pavement life cycle.

Figure 2 shows that the use phase is the main source of global warming due to the significant amount of daily traffic operated for decades and petroleum-driven vehicles. Compared to the other phases, the construction and maintenance phases have less impact on the environment, with the maintenance phase more impactful than the construction phase [75,76]. There is a need to control the emissions from the material phase by using new materials.

**Table 1:** Impact results from life cycle inventory of asphalt binder and asphalt mixture production and materials phases [75,76].

Impact Category	Impact Category Area	Total (per km) for Asphalt Production	Total (per km) for Asphalt Mixture Production	Total (per km) for Material Phase	Contribution of Asphalt Binder and Mixture to Emissions (%)
Depletion of minerals	Asphalt and aggregates	60 tons of asphalt	1000 tons of asphalt and aggregates	1000 tons of asphalt and aggregates	6
Global warming	-	10,382 kg CO_2_-eq	34,420 kg CO_2_-eq	46,147 kg CO_2_-eq	97
Acidification	-	80 kg SO_2_-eq	135 kg SO_2_-eq	216 kg SO_2_-eq	100
Human toxicity	Emissions to air	10,533,660 kg C_6_H_4_Cl_2_-eq	1,054,684 kg C_6_H_4_Cl_2_-eq	11,588,348 kg C_6_H_4_Cl_2_-eq	100
	Emissions to fresh water	33,600 kg C_6_H_4_Cl_2_-eq	33,600 kg C_6_H_4_Cl_2_-eq	67,200 kg C_6_H_4_Cl_2_-eq	100
Eco toxicity	Emissions to air	27,528 kg C_6_H_4_Cl_2_-eq	2738 kg C_6_H_4_Cl_2_-eq	30,266 kg C_6_H_4_Cl_2_-eq	100
	Emissions to fresh water	1320 kg C_6_H_4_Cl_2_-eq	1320 kg C_6_H_4_Cl_2_-eq	2640 kg C_6_H_4_Cl_2_-eq	100

**Table 2:** Environmental impact of material phase of asphalt pavement life cycle across different countries [83].

Studies	Location	Analysis Period (years)	Material Phase Environmental Impact (%)
(Babashamsi et al., 2016) [82]	Malaysia	-	65–75
(Gao et al., 2024) [80]	China	-	>80
(Kayo et al., 2015) [77]	Japan	-	88
(Mendoza et al., 2012) [78]	Spain	>45	48–87
(Praticò et al., 2020) [79]	Italy	-	60–70
(Santero et al., 2011) [81]	US	-	50–60

##### Urban Heat Island (UHI)

UHI is a phenomenon in which heat is accumulated in urban areas due to construction and human activities, posing a threat to urban environments. Asphalt pavements significantly contribute to the UHI effect due to their widespread use, high heat absorption, and the low reflectivity of their dark surfaces. Solar energy that is absorbed by the asphalt binder and stored in the pavement can be reflected in the environment, leading to the UHI effect. A fresh asphalt binder absorbs approximately 95% of sunlight, which is comprised of 43% solar energy, 52% near-infrared light, and 5% UV light, demonstrating significant absorption [84]. The amount of reflected heat is expressed as albedo, which is defined as the proportion of incident light reflected from the pavement surface. In urban areas, UHI can potentially increase the air temperature by between 5 °C and 15 °C [71]. A rapid increase in the average temperature of the environment will create conditions that promote the growth of air pollution [85]. The growing intensity and the adverse environmental effects of UHI demonstrate the need for mitigation measures [86].

#### 4.2.2. Health Effects

Asphalt fumes generated at high temperatures consist of volatile organic compounds (VOCs), polycyclic aromatic hydrocarbons (PAHs), particulates, and greenhouse gases, which are carcinogens. These chemical compounds reduce the quality of surrounding air and adversely affect workers’ health [87]. VOCs, a special group of air pollutants, can have short-term (acute) and long-term (chronic) impacts on human health through inhalation or skin contact, posing a potential health risk to the construction workers [88]. The concentration of VOC ranges between 0 mg/m^3^ and 139.97 mg/m^3^ around the workers. The workers involved in the cleaning of pavement surfaces are at higher risk of cancer, followed by paver drivers and other operators [89].

### 4.3. Economic Issues

**Figure 3:**
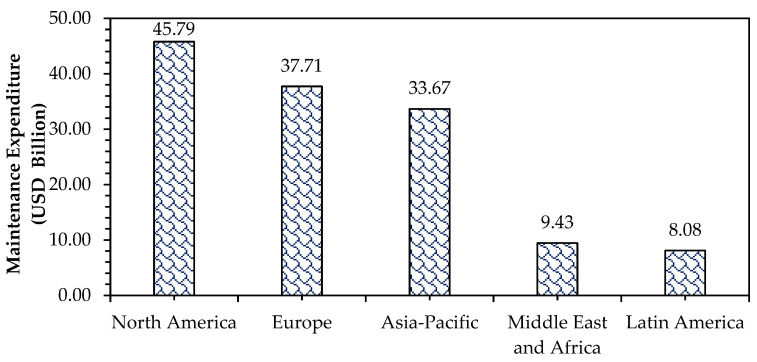
Maintenance expenditure of asphalt pavement across various regions in 2024 [100].

The development of various distress over time reduces the service life of the pavement. To enhance the service life and maintain structural integrity, asphalt pavement is frequently maintained and repaired through resurfacing, crack sealing, pothole repairs, patching, and other rehabilitation strategies. This will result in higher maintenance costs as it involves additional raw materials, manpower and machinery [90]. In 2024, the global road maintenance expenditure of asphalt pavement is valued at USD 150.57 billion and is expected to reach USD 201.95 billion by 2031 at a compound annual growth rate (CAGR) of 3.8% from 2024 to 2031. Figure 3 presents the asphalt pavement maintenance expenditure for various regions in 2024. The large and ageing road network in North America, with high traffic volumes, extreme weather conditions, and rising labor and material costs, requires frequent repairs and maintenance, leading to higher maintenance expenditures compared to other regions [91]. In contrast, the low importance for maintenance, focus on new construction over maintenance, and budget constraints resulting in the lowest maintenance expenditure value for Latin America [92,93,94].

Life cycle costing (LCC) is a systematic economic evaluation method to estimate the life cycle costs of asphalt pavements [95]. Energy and time are the two components that are used for estimating the construction and rehabilitation costs of asphalt pavement in the life cycle cost analysis (LCCA) method. Asphalt binder and mixture production account for more than 50% of the total costs for construction and rehabilitation activities, which involve fuel consumption [96]. A 1% change in the fuel price index leads to a 0.46% change in the asphalt price index, increasing the construction and maintenance costs of asphalt pavement [97]. The increasing costs of asphalt binder and aggregates cause a burden on highway budgets, necessitating alternative cost-effective methods for construction and rehabilitation [98]. Moreover, incorrect selection of binder grade for a specific location can increase the maintenance costs by between 38% and 64% [99].

Additionally, the variations in temperature and precipitation patterns due to extreme climate events aggravating the pavement distresses. Neglecting these effects of climate change in the current pavement design methods may result in underestimation of pavement performance and increased operational and maintenance costs [101]. Each degree Celsius increase in the temperature annually could lead to an extra USD 650 million to USD 700 million annually for the maintenance and rehabilitation of asphalt pavements in the US. This indicates that the costs incurred due to climate change are high during the maintenance and end of life phases, requiring road agencies to allocate additional budgets for effective climate adaptation [62]. As a mitigation measure against warming temperatures, increasing the asphalt layer thickness by 7% to 32% can enhance the pavement service life, though this is not cost-effective [102].

## 5. Currently Adopted Measures to Address the Challenges of Asphalt Binder

The incorporation of different materials in the asphalt binder for enhanced performance can be categorized in three ways based on the percentage of replacement: modifier if the replacement is less than 10%, extender if the replacement is between 10% and 75%, and alternative if the replacement is more than 75%. Various modifiers and alternatives to mitigate the challenges of asphalt binder are discussed in this review.

### 5.1. Modifiers to Enhance Performance of Asphalt Binder

Mitigating distresses in asphalt pavements necessitates an integrated approach that focuses on best practices throughout the pavement’s entire life cycle, from design to rehabilitation. The application of modifiers in asphalt binders to obtain better performance to mitigate different pavement distresses related to various climatic conditions has been analyzed in recent years [103,104].

#### 5.1.1. Polymer Modifiers

Modification of asphalt binders with polymers enhances the performance and service life of asphalt pavements by creating a chemical interaction between the binder and the polymer [105]. Polymer modification is the process of adding polymers to the asphalt binder by mechanical mixing. Thermoplastics such as PE, PP, and PET, together with plastomers like EVA and EBA, as well as elastomers including SBS, SIS, and SEBS are commonly utilized in the binder modification process. Table 3 provides a comparison of studies that have been conducted on the modification of asphalt binders using polymers. Detailed information on the types of polymers used, the mixing technique, i.e., dry or wet, and the most important findings are included in each row, which represents a distinct comparison. High-temperature performance such as rutting resistance and high-temperature grade, low-temperature performance such as cracking resistance, and other pertinent observations such as moisture resistance and cost savings are the categories that have been considered to present the findings to facilitate clarity.

While researchers reported improvements in high-temperature properties in most of the studies depending on the polymer type and concentration, the improvement varied significantly. Yao et al. (2022) [106] identified improvements using PET, while Revelli et al. (2023) [107] presented the positive impact of low-density polyethylene (LDPE), SBS, and WCO for high-temperature grade. However, inconsistent findings are reported by researchers for low-temperature performance of polymer modified binders. Improved low-temperature cracking resistance is reported incorporating PE by Padhan and Sreeram, (2018) [108], whereas Revelli et al. (2024) [109] identified that insignificant change was observed in thermal cracking resistance at −18 °C using PS and PET. Alternatively, in another study, Revelli et al. (2023) [107] found a negative impact of LDPE, SBS, and WCO combination at lower temperatures. These variances in low-temperature performance highlight the complicated interaction between polymer type and asphalt binder.

Improved moisture resistance [110] and fatigue damage resistance [111] were noted in several studies. Alemu et al. (2023) [112] focused mainly on improved indirect tensile strength and reduced rutting occurrence. Conversely, the study by Yao et al. (2022) [106] uniquely quantified the significant economic benefits including cost and GHG emissions reductions associated with their polymer modification approach.

In addition to that, the performance of the modified binder also depends on the binder modification process and is strongly linked to polymer type and concentration. In short, adding polymers to asphalt generally makes it better in terms of performance at high temperatures and moisture resistance. However, the outcomes depend on polymer type, dosages, and mixing methods. Further research is needed to figure out exactly how all these things work together through complete understanding of the interactions between polymers and binders.

#### 5.1.2. Nanomaterials

Nanomaterials have gained immense attention in the last few years due to their benefits in reducing rutting, fatigue cracking, moisture damage, short-term and long-term ageing, and temperature-induced stresses in asphalt mixtures. Nanomaterials such as organo-modified nano clay (OMNC), silica (SiO_2_), zinc oxide (ZnO), calcium carbonate (CaCO_3_), aluminum oxide (Al_2_O_3_), iron oxide (Fe_2_O_3_), titanium dioxide (TiO_2_), and carbon nano tube (CNT) are commonly used for modifying the asphalt mixtures. The larger specific surface area of nanomaterials helps in achieving a uniform and homogeneous distribution in the asphalt binder matrix, which increases the average mean path travelled by lighter-molecular-weight components of the asphalt binder, eventually creating a hindrance to oxidation [113]. As a result, nanomaterial-modified binders show improved oxidation resistance.

From several studies, it has been found that the addition of nanomaterials such as OMNC, ZnO, and SiO_2_ enhances the short-term ageing resistance potential of the modified asphalt binder [114,115,116,117,118], whereas CNT was found to be effective in both improving the short and long-term aging resistance potential (Zhu et al., 2017) [119]. Although the literature indicates that the addition of nanomaterials can dramatically improve aging resistance potential, further exploration of the nanomaterial-modified asphalt binder matrix through Atomic Force Microscopy (AFM) and Fourier Transform Infrared Spectroscopy (FTIR) methods to better understand the effects of various nanomaterials on the aging-resistance potential of asphalt binder is needed [113].

In Table 4, different types of nanomaterials and their effect on the asphalt binder performance, i.e., ageing resistance, moisture damage resistance, intermediate temperature cracking, rutting at high temperatures, and cracking related to low temperatures, are identified. Along with the change in aging resistance mentioned in the previous section, improvements in moisture damage resistance potential were also observed by researchers when nanomaterials were added to the asphalt binder [113]. These improvements were quantified through Energy Ratio (ER) values from the SFE approach and Tensile Strength Ratio (TSR) values obtained from indirect tensile strength (ITS) tests. Based on the outcomes from studies, it has been identified that the addition of nanomaterials like ZnO, Al_2_O_3_, OMNC, and CaCO_3_ has been found to significantly enhance the moisture damage resistance potential of asphalt binder where various aggregate types, including limestone, granite, basalt, sandstone, and quartzite, were considered [115,117].

With a focus on minimizing rutting failure in asphalt binder which is caused by high temperatures, researchers have evaluated the effects of nanomaterial additives such as OMNC, CNT, and nano-TiO_2_ on stiffness values and resistance to permanent deformation at high temperatures [113]. Additionally, certain nanoparticles have reportedly improved low-temperature capabilities according to studies. For instance, CNT additives have demonstrated significant improvements in stiffness values with higher modulus and specific surface area. Comparative studies have presented a correlation between CNT content and stiffness enhancement in asphalt binder [120]. Santagata et al. (2012) [121] found that increasing CNT content from 0.5% to 1% by weight reduced asphalt binder stiffness. However, when CNT concentration rose from 1.5% to 2.25% by asphalt binder weight, the stiffness value fell according to the study conducted by the authors of reference [122].

Based on the existing studies, it can be stated that different nanomaterials have significant potential for enhancing asphalt binder performance by mitigating various distresses, including rutting, fatigue cracking, and moisture damage, across various climatic conditions. While studies demonstrate improvements in oxidation resistance and short- and long-term aging resistance with several nanomaterials, e.g., OMNC, ZnO, SiO_2_, and CNT, the modifier type, concentration, and mixing methods remain unclear. Further research, particularly using AFM and FTIR, is needed to elucidate the complex interactions between nanomaterials and asphalt binders.

**Table 3:** Different types of polymer modified asphalt binder studies with findings.

Source	Type of Plastic	Mixing Method	Findings
(Alemu et al., 2023) [112]	PET and crumb rubber	Dry mixing	Improved indirect tensile strength and reduced rutting potential.
(Yao et al., 2022) [106]	PET	Wet mixing	Improved rutting, fatigue, and stripping performance. During the 50-year analysis period, costs can be reduced by 26% and GHG emissions can be reduced by 29%.
(Revelli et al., 2024) [109]	PS and PET	Dry mixing	Improved resistance to moisture damage by 11% with addition of 9% PET and 3% PS. Improved rutting resistance by 150–190%. Reduced intermediate temperature cracking resistance by 14% with addition of 3% PET and 37% with 3% PS. Change in resistance toward thermal cracking at −18 °C was insignificant.
(Padhan & Sreeram, 2018) [108]	PE	Wet mixing	Increased stiffness with reduced penetration by 25%. Improved the low-temperature and fatigue properties by 14% at −18 °C and 250% at 58 °C, respectively.
(Revelli et al., 2023) [107]	LDPE + SBS + WCO	Wet mixing	Improved high-temperature grade for both SBS- and plastic-modified binders. Presented negative impact at low temperatures with an increase in low-temperature performance grade.
(Yin, F. et al., 2023) [111]	LDPE + RET and SBS	Wet and dry mixing	Improved rutting resistance by 70%. Increased fatigue damage resistance of asphalt binder by 30%.

#### 5.1.3. Rejuvenators

As recycled asphalt pavement (RAP) is cost-effective and ecologically beneficial, it has become increasingly popular in road development and repair [123]. Conversely, one of the key issues with RAP is that asphalt additives become less valuable over time as they age and harden. Several studies have used WCO, waste engine oil (WEO), and waste vegetable oil (WVO) as well as other waste oils to make asphalt binder modification work better again [124,125,126,127]. Food preparation generates WCO that has been identified for use in repairing old asphalt’s characteristics [128]. Likewise, WEO produced by lubrication of vehicle engines has shown to be a potent rejuvenating agent [129]. Similar potential as an efficient rejuvenator in asphalt binder modification is shown by WVO, generated from culinary operations using plant oils [130].

Table 5 shows some studies where WCO, WVO, and WEO have been used for asphalt rejuvenation. The results indicate varied impacts on asphalt qualities; some studies show increases in viscosity, ductility, cracking resistance, and temperature resilience [125]. A study by Dokandari et al. (2017) [126] reported vegetable oil as superior for softening stiff RAP materials after comparison of the performance of vegetable oil and engine oil. Devulapalli et al. (2019) [131] highlight improved tensile strength and moisture resistance using WVO, while the authors of reference [127] identified reductions in rutting factors and improvements in low-temperature performance with WEO. The table indicates that waste oils show promise as sustainable alternatives for asphalt rejuvenation, yet their effects are highly dependent on the specific oil type and the method of incorporation into the asphalt mixture.

Including waste oils as rejuvenators in asphalt manufacture offers a lot of measurable financial and environmental advantages. The use of RAP combined with waste oils can lead to a reduction of approximately 12% in CO_2_ emissions and 15% in energy consumption during asphalt production processes when comparing warm mix asphalt (WMA) technologies that utilize RAP with traditional methods [132].

**Table 4:** Studies on nanomaterial-modified asphalt binder performance.

Source	Type of Nanomaterials	Performance of Nanomaterial Modified Asphalt Binder				
		Aging Resistance	Moisture Damage Resistance	Intermediate Temperature Performance	High Temperature Performance	Low-Temperature Performance
(Ashish et al., 2017 [114]) (I) (Hossain et al., 2014 [115]) (II) (You et al., 2011 [116]) (III)	OMNC	Improved short term aging resistance by 30% at 90 °C (I)	Reduced moisture resistance by 31% (II)	Increased load cycle to failure (I)	Decrease of 83% in non-recoverable creep compliance with improved recovery, and increased rut factor by 300% at 64 °C (I, II)	Decreased failure strain by 45% at −18 °C making susceptible to thermal cracking (III)
(Zafari et al., 2014 [117]) (IV) (Shafabakhsh et al., 2014 [133]) (V) (Zhang et al., 2015 [118]) (VI) (Hamedi et al., 2015 [134]) (VII)	SiO_2_, TiO_2_, ZnO, CaCO_3_, Al_2_O_3_, and Fe_2_O_3_	Improved aging resistance potential by 25% (IV)	Improved moisture damage resistance potential by 30% to 60% (VII–VIII)	Prevents cracks generation and propagation with increased load cycle to failure (V) [133]	Decreased permanent deformation at 250 kPa and 60 °C by 30% (V) Improved viscosity and softening point and decreased ductility (VI)	N/A
(Zhu et al., 2017 [119]) (VIII) (Ameri et al., 2016 [135]) (IX) (Xiao et al., (2011) [120]) (X) (Santagata et al., 2012 [121]) (XI)	CNT	Improved short-term and long-term aging by 25% and 53%, respectively, at 50 °C (VIII)	N/A	Improved fracture behavior and increased fatigue life (IX) [135]	Improved resistance to permanent deformation by 55% at 64 °C (X)	Creep stiffness and m-value found to be statistically similar to control binder (XI)

**Table 5:** Studies on WCO, WVO, and WEO with findings.

Source	Type of Rejuvenator	Mixing Method	Findings
(Li et al., 2022) [125]	WEO and WCO	Wet mixing	WEO increased asphalt viscosity by 25.7%, while WCO decreased it by 10.4%. Both additives improved ductility, cracking resistance and temperature resilience.
(Dokandari et al., 2017) [126]	WEO and WVO	Wet mixing	Vegetable-based oil performed better than engine oil in terms of softening the stiff RAP materials. Improved temperature resistance and enabling up to 80% recycled asphalt pavement incorporation.
(Devulapalli et al., 2019) [131]	WVO	Dry mixing	Improved moisture damage resistance. Decreased stiffness, and allowed an increase in the RAP content.
(Chen, Hu et al., 2021) [127]	WEO	Wet mixing	Decreased rutting factor by 34.5% at 60 °C and improved low temperature performance by 57% decrease in S (stiffness) value and 19% increase in m-value (creep rate) at −18 °C.

### 5.2. Alternatives to Asphalt Binder

Increases in environmental standards increase and the cost of asphalt binder have prompted researchers to explore alternate binders suitable for asphalt mixtures [136]. Furthermore, consumption of asphalt binder for asphalt paving has increased rapidly throughout the centuries, making it increasingly costly. Additionally, asphalt binder is a major contributor to carbon emissions during the production of asphalt mixtures. Poor quality of asphalt binder results in early failures of pavement, frequent repairs, high maintenance cost, and decreases service life of the pavement. Hence, there is an urgent need to identify alternative sustainable and cost-effective materials to asphalt binders to address these issues, especially if at least 10% replacement of the binder can be achieved [137].

In recent decades, the European Union (EU) has implemented initiatives aimed at mitigating environmental impacts, reducing energy and water use, minimizing raw material exploitation, and decreasing GHG emissions [138]. As a result, the road construction industry has been advancing towards more environmentally friendly and sustainable practices, advocating for the incorporation of alternative materials such as waste or secondary raw materials in asphalt mixtures [139,140].

Natural asphalt (NA) has gained significant attention recently due to its improved physical and chemical properties, containing sulfur content of 40% and nitrogen content of 80% more than conventional asphalt binder, making it suitable for incorporation in asphalt pavement construction. The moisture resistance of NA mix was found to be 0.37% more than that of the AC (40–50) mix [141]. Among the naturally available asphalts, rock asphalt (RA) and Gilsonite and Trinidad lake asphalt (TLA), RA is highly compatible with asphalt binder without chemical processing. RA in its natural form is impure and unsuitable for asphalt mixture production. RA subjected to heat treatment (163 °C for 20 h) showed improved moisture resistance, durability, and high resistance to temperature susceptibility [142]. Due to high mineral and asphaltene content, TLA is hard to employ as a substitute for asphalt binder in asphalt mixture. TLA-modified SBS improved the fracture resistance, ageing resistance, and service life of asphalt pavement. Gilsonite improves the high-temperature performance of the asphalt binder but decreases the low-temperature cracking resistance of the asphalt binder [143].

Buton granular asphalt (BGA), a type of rock asphalt (asbuton—ASB), has been used as a filler or an asphalt binder for asphalt mixtures. Bio-asphalt is a component of bio-oil produced from biomass through the pyrolysis of coconut shells (BioCS). Bio-asphalt and asbuton asphalt binder are together called bio-asbuton [144]. A combination of 15% ASB (by weight of mixture) and 25% BioCS (by weight of ASB) can be used as an alternative binder to Pen 60/70 in asphalt mixtures [145].

Bio-asphalt like vegetable resin (VR), when mixed with additives like SBS, polyethylene wax (PEW), and waste olive oil (WOO) with lower oil/resin ratios (0.35 O/R), can be used in conventional asphalt mixtures, whereas higher ratios (0.55 O/R) may be used as a rejuvenator for mixes with high percentages of RAP [136]. A study by the authors of reference [136] found that WCO improved the thermal cracking resistance by 107% and moisture resistance by 4% and decreased the fatigue resistance by 40% and rutting resistance by 70% of asphalt mixtures at 30% addition with PG 58-28.

Swine manure as a bio-binder improved the low-temperature performance of asphalt binder, decreased energy consumption by reducing the mixing and compaction temperatures, and reduced the construction cost by 75% [146]. Additionally, Musco et al. (2024) [147] found that the use of swine manure can reduce the global warming potential by 7.8%.

The use of recycled materials, such as re-refined engine oil bottoms (REOBs), a petroleum-based asphalt binder, exhibited similar mechanical performance to conventional HMA mixes in terms of moisture damage resistance, tensile resistance, and stiffness [17].

### 5.3. Mitigation Measures for Environmental Burdens

Various methods are available to reduce the environmental impacts of asphalt pavements throughout the life cycle. Mitigation measures involving recycled materials, alternate construction practices in asphalt mixture production for lowering GHG emissions, and cool pavements for minimizing UHI effect are discussed in the subsequent sections.

#### 5.3.1. GHG Emissions

LCA studies have shown that asphalt binder and mixture production significantly contributes to GHG emissions and can be minimized by using materials that have a lower carbon footprint than asphalt binder and changing the asphalt mixture production techniques. Incorporation of recycled materials and adaptation of technologies like WMA can reduce emissions.

##### Recycled Materials

One of the most effective mitigation measures to reduce GHG emissions during the material phase is through incorporation of recycled materials such as RAP and recycled asphalt shingles (RAS). The asphalt mixtures prepared with 50%, and 20% RAP reduced the cradle-to-gate GHG emissions by 30% and 12%, respectively, assuming the same asphalt binder content of RAP and mixtures. The use of RAP reduced GHG emissions by 2.6 MMT kg CO_2_-eq in 2021 [148]. RAP mixes with waste oils as rejuvenators can reduce GHG emissions by approximately 12% and energy consumption by 15% when compared to WMA [132].

RAS contains more than 15% of asphalt binder that are stiffer than the commonly used binders in paving. GHG emissions from cradle-to-gate phase are reduced by 4% and 10% for asphalt mixtures containing 2% and 5% RAS, with recycled binder ratios of 8% and 20%, respectively [149].

##### Warm Mix Asphalt (WMA)

Numerous studies have explored alternative construction techniques aimed at lowering the production temperatures of asphalt mixtures to reduce potential carbon emissions [150]. WMA technology reduces the production temperature of asphalt mixtures by using water or additives that decrease the viscosity of asphalt binder [151]. A 10 °C reduction in the production temperature of asphalt mixtures can reduce the carbon emissions by 1 kg/ton [152]. WMA technology lowers the asphalt mixture temperature by 50 °C to 70 °C and the energy consumption by about 5% to 13% compared to hot mix asphalt (HMA) mixtures [153,154]. The GWP of WMA mixes is about 75% of HMA due to lower energy consumption and reduced emissions during production [63]. Integration of RAP with WMA can achieve a decrease of up to 12% in carbon emissions, 15% in energy consumption, and 15% in water use [155]. Mitigation measures have been developed to address the environmental impacts of asphalt pavements, which are discussed in the subsequent sections.

#### 5.3.2. Cool Pavements for UHI Effect

The United States Environmental Protection Authority (USEPA) defined cool pavement as paving materials that have been modified to reduce temperature compared to conventional pavements. Various approaches, for instance, reflecting pavements, evaporative and water-retaining pavements, and thermochromic or phase change materials (PCMs) incorporated in asphalt pavements, have been devised to provide cool pavements in response to the issues related to the elevated temperatures of asphalt surfaces. Despite numerous studies aimed at mitigating the effects of UHI, nearly all are predicated on three principles: incorporating water inside the pavement, enhancing the reflectivity of pavements, and modifying the thermal conductivity of pavements using highly conductive or insulating materials [25,63,136,156]. When developing cool pavement, it is vital to have a solid understanding of the thermophysical properties of the materials used in the pavement. Some of the influential parameters that have been usually considered to determine heat storage and heat transfer in pavement materials are thermal diffusivity, thermal emissions, density, heat capacity, thermal conductivity, solar reflectance index, and albedo [157].

Evaporative and water-retaining pavements cool the pavement through water evaporation by replacing the fines with porous materials [158]. The increase in air voids due to the reduction in fines enhances the water permeability and reduces thermal conductivity [159]. Porous and permeable pavements have high thermal insulation and low heat storage properties due to high air void content, keeping the pavement cooler at night and warmer during the day [160]. Water-retaining pavements have high absorption and low permeability, holding water at the top and subbase layers and remaining cooler than evaporative pavements for long time [161].

Reflective pavements are a cost-effective and simple method to mitigate the UHI effect by increasing the albedo through reflective coating, which reduces the maximum daily surface temperature. A study by Synnefa et al. (2011) reported that increasing the albedo of 1250 km^2^ of pavement by 0.25 could lead to a decrease in air temperature of approximately 0.6 °C, resulting in reduced energy demand and lower ozone concentrations [162,163]. Resurfacing asphalt pavements using light-colored sealing compounds is a typical practice for increasing the pavements’ reflectivity as they improve the albedo [138]. An aged pavement is covered with a thin coating of asphalt binder, and then fine aggregates are spread out over it to create this sealed surface. While reflecting pavements can reduce surface temperatures in summer, they further lower the temperature during winter, which is undesirable.

Thermochromic materials such as vanadium dioxide (VO_2_), TiO_2_, and ZnO change their color and properties with temperature fluctuations. During summers, the thermochromic materials absorb lower solar energy and more solar energy is captured during winters [162,163]. The modifiers can reduce the asphalt mixture surface temperature by 6 °C during summer and increase it by 3 °C during winter compared to conventional binder [164].

PCMs in the asphalt mixture prevent the pavement from attaining high temperatures due to climate change and the heat island effect. PCMs are substances that absorb significant amounts of energy known as latent heat of fusion from their surroundings during the melting process while maintaining a constant temperature [165]. PCMs such as paraffin waxes, fatty acids, polyethylene glycols (PEGs), and salt hydrates have been utilized in asphalt mixtures. The type of PCM plays a significant role in improving the thermal performance of the asphalt mixture. PEG-2000 and PEG-4000 are widely used due to their suitable phase change temperature range, chemical stability, and non-toxicity. PEG-2000 and PEG-4000, encapsulated with SiO_2_, enhanced the thermal performance of the asphalt mixtures by reducing the mix temperature by 9 °C while maintaining similar mechanical performance to the conventional HMA mix [156].

## 6. Limitations of Alternative Solutions

Modifiers or alternatives to asphalt binders present various challenges, including performance, environmental impact, and cost, in addition to their benefits, and these are outlined in the sections below.

### 6.1. Performance Issues

Modified asphalt binders incorporate several additives, e.g., polymers, nanomaterials, and recycled materials, to improve their performance traits. Notwithstanding their advantages, modified asphalt binders present major difficulties over their lifetime, i.e., during manufacture, storage, application, and service periods. Their long-term performance is influenced by factors like material compatibility, manufacturing complexity, environmental effects, and physical aging process. The section below covers the difficulties related to the modified asphalt binders in several phases of the life cycle.

#### 6.1.1. Material Variability

Variability in materials can influence the performance of modified asphalt binders. The behavior of the base binder under various loading and environmental situations can be greatly altered by the interaction with the modifiers.

Using waste plastics in asphalt mixes is challenging due to worldwide diversity in sources of plastic. Due to this non-uniformity of plastic-modified binders, field level execution is being hindered. Although crystalline (*X*c) and viscosity are found to be two effective parameters to categorize plastics instead of source, further research is required to better understand the coating efficiency on hot aggregates and binding to filler characterization of polyethylene. PEs with an (*X*c) value less than 60% are found to behave as a binding agent, while those with higher (*X*c) values show an increased tendency to behave as a filler (Revelli et al., 2024b) [166]. Similarly, the addition of SBS improved the elasticity and temperature sensitivity of asphalt binder. However, their effectiveness is affected by the type and concentration applied, as variability in polymer sources can lead to differences in performance characteristics [167]. Furthermore, the use of nanomaterials can improve asphalt binders’ performance; however, changes in the quality and dispersion of the nanoparticles might result in different performance [168]. Additionally, waste oil rejuvenators’ effects depend on the oil type, mixing efficiency, and potential contact with asphalt binder chemicals.

#### 6.1.2. Mixing Methods

To obtain the optimized performance of modified asphalt binders incorporating different additives, their mixing method is one of the important parameters which involves several challenges that must be carefully addressed.

Polymers, particularly SBS, SBR, EVA, and plastics, are widely used to modify the asphalt binder by adopting specific mixing methods individually. There are two different ways to mix plastic into asphalt pavements: wet mixing and dry mixing. In the wet mixing method, asphalt binders are blended with plastic before they are mixed with aggregates. Because of variation in densities, asphalt and plastic do not mix well in the wet mixing process, resulting in phase separation, where plastics tend to float to the top of the asphalt binders when the mixture is kept at a high temperature for a longer duration [110]. Alternatively, in dry mixing, the plastic is added directly into the aggregates and asphalt binder without the need for extra equipment for pre-binder blending [169]. Joy et al. (2024) [170] indicated that elevated coating temperatures positively affected the aggregate coating with plastics via a reduction of 2% in permeable pores volume for 5 °C increments. However, the uniformity and consistency of plastic-modified mixtures in the dry mixing method varies based on the interaction between the plastic, aggregate, and asphalt binder while coating the aggregate.

Similarly, achieving efficient dispersion is also one of the main difficulties in including nanoparticles in the asphalt matrix [171]. Strong van der Waals between nanoparticles cause agglomeration inside the modified asphalt binder matrix, a frequent problem preventing their equal distribution and resulting in uneven performance [172]. Furthermore, careful examination is needed throughout the mixing operations to prevent too-strong shear from destroying the structure of the nanomaterials, therefore compromising performance advantages. Moreover, variations in the nanomaterial-modified binder might be due to the mixing equipment and the necessity of certain circumstances, like high temperatures [168].

Incorporating waste oils as rejuvenators into modified asphalt binders also comes with challenges concerning the mixing conditions and the characteristics of the waste oils themselves. WVO, WEO, and WCO are commonly used as rejuvenators. Each form of waste oil might vary greatly in terms of viscosity, density, and chemical structure, therefore complicating its incorporation into asphalt binder [173]. Obtaining a homogeneous mix requires careful assessment of the properties of the oil and the condition in which it is mixed with the asphalt binder. Lower compatibility might lead to issues including unequal aging and poor asphalt matrix performance [173].

#### 6.1.3. Dosages

The variations in the dosages of the modifiers while modifying the asphalt binder can lead to significant challenges in terms of performance. Understanding these challenges requires quantification of how different dosages affect the overall effectiveness of modified asphalt binders to identify the optimal dosages for each type of modifier under specific climatic conditions.

Polymers such as SBS are widely used to improve asphalt’s elasticity and temperature resistance. Performance studies show that adding 5% SBS generally yields a substantial enhancement in rutting resistance, with the G*/sin δ value increasing by 60% compared to the neat asphalt [174]. However, if the dosage exceeds 7%, the modified binder may exhibit increased stiffness, which will lead to lower ductility and low-temperature cracking resistance. Moreover, at dosages over 10%, a 40% reduction in fatigue life is often observed for modified asphalt mixtures compared to those with optimal content of polymer [175]. Similarly, in the case of modification by plastics, although ideal ratios vary with environmental circumstances and need thorough investigation, plastic content in asphalt such as 9% PP is recommended by Li et al. (2022b) [125] to improve viscosity and high-temperature performance. Two very commonly used plastics in wet blending process are LDPE and high-density polyethylene (HDPE). The dosage of these recycled plastics varies from 1% to 12% by weight of asphalt binder [111]. However, research has selected smaller doses of plastics between 1% to 3% considering the wet mixing process to prevent phase separation issue [127]. Consequently, identifying the optimal dose for best performance is still difficult as the effects on the mechanical characteristics of the binder differ.

Studies have indicated that incorporating nano-SiO_2_ significantly enhances the viscosity and softening point of asphalt binders. Asphalt binder modified with 4% nano-SiO_2_ exhibited a 28% increase in viscosity compared to neat binder. On the other hand, increasing the dosage beyond optimal levels can yield adverse effects, e.g., exceeding the dosage of nano-SiO_2_ beyond 4% led to increased agglomeration, which negatively impacted performance and workability, resulting in a reduction in penetration by approximately 35% compared to the control sample [176]. The difficulty is in adjusting the dose to get intended improvements without causing agglomeration of nano modifiers in the asphalt matrix.

As a rejuvenator, waste oil is usually utilized at lower percentages. Devulapalli et al. (2019) [131] found that tensile strength was maximized by 6% WVO by weight of the binder, thus improving moisture resistance. Comparably, Dokandari et al. (2017) [126] discovered that 5.4% of WEO enhanced flow values. Research highlights that the addition of waste cooking oil at a dosage of 5% improves low-temperature performance and reduces mixing and compaction temperatures by up to 15 °C [177]. However, when the dosage exceeds 7%, the rutting resistance of the rejuvenated asphalt mixture decreased by almost 35% [178]. This decreased performance highlights the difficulty in finding a good combination while preserving the structural integrity of the binder under heavy loads and improving workability. Moreover, overdosed waste oils might cause a change in the rheological characteristics of the asphalt binder. High-oil-content compositions can reduce shear strength, causing early failure in very traffic-heavy locations (roadways with >30 million ESALs) [177].

#### 6.1.4. Construction

Binder modification in construction practices presents challenges that must be carefully managed. This section presents the primary challenges associated with the construction practices of using nanomaterials, polymers, waste oils, and other additives in modified asphalt binders.

As mentioned in the previous section, proper dosage is critical; if the amount of polymer is excessive at the time of preparation at large scale, it may lead to a binder that is too rigid, consequently impairing its low-temperature performance. Conversely, insufficient polymer content may fail to provide the desired performance that the modified asphalt requires to perform effectively under load and high temperature [105]. Moreover, ensuring proper mixing and blending of polymers with asphalt binder is also vital. The quality control of polymer sources is another challenge during construction which can make binder’s performance inconsistent [179].

For nanoparticles, one major challenge is the agglomeration during the mixing process, which can hinder their uniform distribution in the asphalt matrix [171]. Such uneven dispersion can result in localized weaknesses within the asphalt mixture, ultimately compromising its strength and durability [180]. Moreover, the variability in the preparation methods, e.g., different mixing techniques, mixing durations, and dispersion methods, of nanomaterial-modified asphalt can lead to inconsistencies in performance.

One significant challenge during construction is maintaining the right balance of waste oil in the mix [173]. Additionally, improper dosage levels can lead to reduced temperatures at which the asphalt can be applied, affecting the overall workability and performance of the mixture during and post construction [181].

#### 6.1.5. Long-Term Performance

Each modification type brings specific challenges during their use-stage. These challenges can significantly influence long-term performance, necessitating thorough examination of field performance.

Research indicates that while a 5% SBS modification can improve fatigue resistance by up to 50% initially, as the asphalt ages, there is a noted decline in performance fatigue, which can decrease by as much as 40% when the SBS dosage exceeds optimal levels [182,183]. The long-term performance data indicate that nanomaterials, e.g., Al_2_O_3_, SiO_2_, and TiO_2_, can increase tensile strength by as much as 20% to 30% compared to neat asphalt [184]. However, durability concerns emerge when assessing performance over time; there is evidence suggesting that aged asphalt modified with nanomaterials may experience a 15% to 25% reduction in performance metrics such as fatigue resistance, especially when subjected to high-moisture conditions [185].

Studies show that waste oils can provide short-term benefits in terms of reducing viscosity and improving workability by up to 15% during initial application [186]. However, substantial long-term durability issues may occur; for instance, asphalt mixtures incorporating waste oil have been observed to exhibit up to a 30% to 50% reduction in rutting resistance over time compared to virgin asphalt counterparts, particularly in high-temperature conditions, leading to potential road surface failure [187]. In another study, it was reported that the mixing and compaction temperature of asphalt mixture with WCO are reduced by up to 15 °C. However, the rutting resistance and fatigue resistance of modified asphalt binder and mixture with WCO are compromised [177].

### 6.2. Environmental Impact

The shift towards modified asphalt binders necessitates careful consideration of the environmental factors associated with the modifiers. Understanding environmental considerations is critical for ensuring the sustainability of asphalt infrastructure.

The production of synthetic thermoplastic elastomers, e.g., SBS, is often reliant on petrochemicals, which can contribute to greenhouse gas emissions and resource depletion [188]. Concerns have also been raised about the temperature stability and durability of plastic-modified asphalt binders during the use stage. As polymers break down due to natural age, they may release microplastics into the environment, which could be harmful to wildlife and the health of the ecosystem as a whole [166]. Moreover, another alarming factor was identified by Thomus L. Junod (1976) [189] in a NASA report, namely that PE, PP, EVA, ethylene-butyl acrylate (EBA), SBS, styrene-isoprene-styrene (SIS), SBR, and styrene-ethylene/butylene-styrene (SEBS) can all produce toxic gases when burned, with the potential to release harmful pollutants like dioxins, furans, carbon monoxide, and volatile organic compounds into the air, posing potentially significant health risks. As fire hazards become increasingly common, research engineers and scientists must consider multiple parameters before selection of modifiers. Rahman et al., in 2013 [190], examined the use of waste polyethylene and PVC in hot asphalt mixtures, noting that while these materials are biologically non-degradable and pose significant environmental challenges, their incorporation into asphalt can yield properties that meet acceptable performance standards. Additionally, recycled PP-modified binders have been identified as having 12% and 5% more environmental impact due to maintenance and additive-related effects, respectively, showing more impact than traditional binders [191]. Another commonly used modifier, SBS, improves aging resistance under salt environments but requires attention to temperature sensitivity and degradation mechanisms [192]. So, it is crucial to concentrate on developing polymers that enhance asphalt performance while also being environmentally sustainable in terms of biodegradability and recyclability.

One of the primary environmental concerns with the use of nanomaterials is their potential toxicity. The small size of nanoparticles allows for easy migration through ecosystems, which can lead to unexpected interactions with biological systems and possible bioaccumulation [193]. Studies indicate that certain nanoparticles may have detrimental effects on aquatic and terrestrial organisms, such as negative effect of CNTs on aquatic organisms, like disrupting cellular membranes in daphnia magna (water fleas) and inhibiting growth in algae (*Chlorella vulgaris*), and on terrestrial organisms, such as reducing soil microbial diversity and earthworm (*Eisenia fetida*) viability, raising valid concerns about their long-term environmental safety [194]. Furthermore, the synthesis processes in the production and disposal of nanomaterials often require significant energy and resources, contributing to a larger carbon footprint than traditional materials [195]. Additionally, contaminants present in waste oils, such as heavy metals, solvents, and other hazardous substances, can negatively impact the properties of the asphalt binder and pose environmental risks such as leaching underneath soil, stormwater runoff transporting contaminants from pavements to waterways directly, and toxic emissions into the ecosystem during asphalt production and placement [14].

### 6.3. Economic Challenges

The economic feasibility of these modifiers is reliant on their LCCA, which evaluates the total cost of ownership over the entire lifespan of a product. The long-term performance data on polymer-modified binders from the literature indicate that the performance of modified binders may reduce with time due to the challenges mentioned above. This could result in asphalt pavements requiring significant maintenance or refurbishment approximately 5 to 7 years earlier than anticipated, particularly under heavy traffic conditions. The resulting costs for premature repairs can escalate to USD 25,000 or more per mile, highlighting the economic consequences tied to dosage mismanagement [196,197]. Additionally, environmental factors such as thermal variations and UV exposure can lead to stiffness increases and a corresponding reduction in ductility, creating costly challenges in maintaining asphalt pavement integrity, especially in regions subjected to severe climatic conditions such as prolonged high temperatures, freeze–thaw cycles, intense UV exposure, heavy rainfall/flooding, and coastal saline environments.

When considering the LCCA from the literature, the long-term performance benefits of nanomaterial modifications must be weighed against their higher initial costs. For instance, using 2% nano-SiO_2_ can enhance the fatigue resistance of modified asphalt by approximately 20% compared to conventional binders [198]. However, long-term performance data indicate that aging of nanomaterial-modified asphalt can lead to reductions in effectiveness of 15% to 25% over time, costing an estimated USD 10,000 to USD 30,000 per mile for repair or resurfacing within the first 5 to 7 years of service [199]. Another challenge is the potential for increased costs associated with the careful handling and application of nanomaterials [200]. These materials often require specialized equipment and techniques for their integration into asphalt, which can increase overall project costs and complicate logistics during construction. Also, the variability in the quality of waste oils can lead to performance deterioration, with studies suggesting a potential 30% to 50% decline in rutting resistance compared to standard binders [201]. The required repairs or resurfacing arising from these performance issues can incur costs close to USD 20,000 per mile, especially when asphalt longevity is compromised, leading to the need for replacements within a 5-year timeframe [202].

## 7. Research Gaps and Future Road Map

Despite advances in asphalt binder modification, significant research gaps persist, limiting the widespread use of these modified binders. The key challenge lies in the variability in modifier types, such as plastics, nano material, and waste oils. To address this, the characterization of these modifiers is essential, with a focus on establishing correlations between their properties and the performance characteristics of asphalt binders. Additionally, further research is necessary at the binder chemistry level to explore the interactions between these modifiers and asphalt binders produced from different refinery process methods. This understanding will be crucial for ensuring asphalt binder and modifier compatibility, as well as for practical use of modified binders.

Standardized methods for blending and identification of optimal dosages of these modifiers is required. The limited amount of data available to identify optimal dosages and appropriate mixing techniques for various modifiers affects the performance of modified binder and creates challenges during and after the mixing process. In the wet mixing method, phase separation in the polymer-modified asphalt binder matrix is a major issue, whereas the dry mixing method lacks the uniformity and consistency of plastic-modified mixtures in terms of coating the aggregate properly. The addition of potential stabilizers/compatibilizers to overcome the issues in the wet mixing method and the identification of optimal dosages with effective mixing methods for the modifiers based on laboratory and field-level performance data are needed to recommend standardized methods for blending.

Contemporary research insufficiently examines the efficacy of these modified asphalt binders and alternatives under the sequential impacts of extreme climatic events, including wildfires, abrupt temperature fluctuations, and flooding. Further research is needed, conducting field scale studies and laboratory experiments to simulate the combined effects of wildfires which include heat and chemical exposure, rapid temperature fluctuations, i.e., freeze–thaw cycles, and flooding, which considers prolonged water retainment, on the performance of the modified and alternative asphalt binders. Characterization of these alternatives and modifiers under extreme climatic conditions is an urgent need in order to identify a sustainable solution.

The current study presented limited data on plant-level feasibility for some of these modifiers and alternatives, which is a major concern before going to plant-level production. Several challenges exist in plant-level production, such as quality control of the polymer blending and nanomaterial mixing to avoid phase separation and agglomeration of the modifiers and maintaining the right balance of waste oil. Pilot studies may be required before the spread of further field applications of these modified asphalt binders.

**Figure 4:**
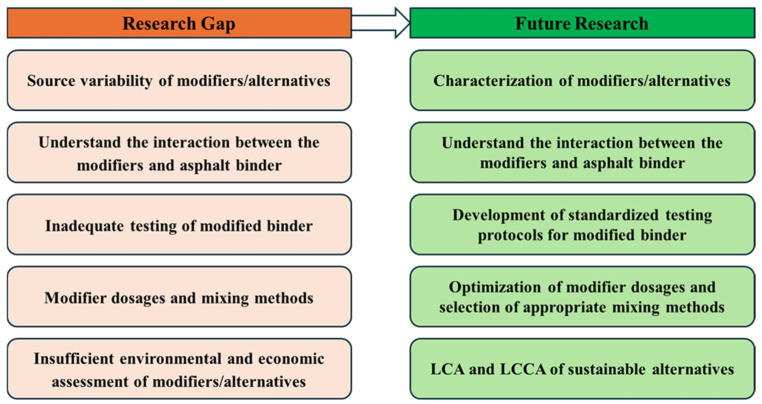
Research gap and future road map.

Data inadequacy is found in evaluating the impact of these modifiers and alternatives on the environment and human health, along with their cost effectiveness. Some of the nanomaterials can spread toxicity to aquatic life by migrating through ecosystems, while others contribute to a larger carbon footprint and breaking down polymers due to natural age and may release microplastics into the environment as well. Contaminants in waste oils, such as heavy metals, solvents, and other hazardous substances, can leach into the ecosystem. Additional leaching tests for modified binders, feasibility analysis after pilot-scale implementation, and life cycle assessment including cost analysis would be recommended to evaluate the economic and environmental benefits of these modifiers and alternatives to better understand their feasibility in large-scale applications. In summary, following the pathway for future research (Figure 4) facilitates widespread practical implementation, establishing these solutions as sustainable alternatives.

## 8. Conclusions

Our roads are failing faster than ever. Premature deterioration, driven by outdated asphalt binder technology, is costing billions in maintenance and repairs, while climate change accelerates the decay. This unsustainable trajectory requires a paradigm shift, not incremental improvements. The critical question is not if we need sustainable alternatives but how quickly can we implement them. To address this, this study aims to provide a comprehensive overview of the challenges associated with asphalt binders while also exploring alternative binders that are cost-effective, environmentally friendly, and performance-enhancing. Additionally, the study examines the limitations of these alternatives and identifies research gaps, outlining a pathway toward the development of more sustainable binders. Based on the outcomes of this study, the following conclusions can be drawn:

- **Performance and environmental issues with asphalt binders:** Asphalt binders, though only 4–7% of the mix, significantly affect pavement life. Poor binder selection and field compaction can reduce service life by up to 30% and increase maintenance costs significantly. Asphalt binder and mixture production are the major sources of greenhouse gas (GHG) emissions, contributing to over 50% of total emissions among the various life cycle phases of asphalt pavements.
- **Enhanced performance of asphalt with the use of polymer and nano modifiers:** Additives like styrene–butadiene–styrene (SBS) and low-density polyethylene (LDPE) can improve the rutting resistance of asphalt binder by up to 200% and fatigue damage resistance by 30%, while nanomaterials (e.g., SiO_2_, TiO_2_, ZnO) enhance aging resistance by 25% to 30% and moisture damage resistance by 30% to 60%. Nanomaterials improve resistance to permanent deformation by 30% and 55% at 60 °C and 64 °C, respectively, though their impact on low-temperature performance is still underexplored.
- **Environmental and economic impact of modifiers:** Implementation of polyethylene terephthalate (PET) can reduce costs by 26% and greenhouse gas (GHG) emissions by 29%, considering a 50-year analysis period. Use of reclaimed asphalt pavement (RAP) combined with waste oils can lead to a reduction of approximately 12% in CO_2_ emissions and 15% in energy consumption during asphalt production of warm mix asphalt (WMA) when compared to traditional methods.
- **Promise of sustainable alternatives:** Bio-based binders reduced global warming potential by 7.8%, and recycled materials including reclaimed asphalt pavement (RAP) and recycled asphalt shingles (RAS) decreased the greenhouse gas (GHG) emissions by 10% to 20%. However, additional research is necessary to evaluate their feasibility, cost-effectiveness, and long-term stability in real-world applications.
- **Need for enhanced characterization and binder compatibility:** There is a crucial need for in-depth characterization of alternative modifiers to understand their interactions with asphalt binders. Further research into binder chemistry and compatibility with various refinery processes will ensure the practical and effective use of modified binders. To enable broader use of modified binders, the development of standardized test methods for blending, dosage optimization, and performance evaluation under extreme climatic conditions is essential. Additionally, comprehensive environmental impact assessments, including leaching tests and life cycle analyses, are necessary to gauge the feasibility and sustainability of these modified binders

### Call to Action

In summary, the asphalt infrastructure that underpins our modern world is a ticking time bomb. With over 50% of material production emissions stemming from asphalt binders and urban heat islands worsening due to their widespread use, the environmental cost of inaction is no longer a distant threat—it is a present-day reality demanding immediate, transformative solutions. While the challenges outlined in this review are significant, they are not insurmountable. However, they require a radical departure from conventional approaches. The time for incremental improvements is over. The development and deployment of sustainable asphalt binder alternatives must be urgently accelerated, backed by substantial investment in research, development, and implementation. The cost of inaction will far exceed the investment required to secure a sustainable future for our infrastructure.

## Data Availability

Data sharing is not applicable, no new data were created or analyzed in this study.

## Abbreviations

The following abbreviations are used in this manuscript:

PE	Polyethylene
PP	Polypropylene
PS	Polystyrene
PET	Polyethylene terephthalate
EBA	Ethylene–butyl acrylate
SBS	Styrene–butadiene–styrene
SIS	Styrene–isoprene–styrene
SBR	Styrene-butadiene rubber
SEBS	Styrene–ethylene/butylene–styrene
WCO	Waste cooking oil
UV	Ultraviolet
UHI	Urban heat island
GHG	Greenhouse gas
ICE	Internal combustion engine
CO	Carbon monoxide
NO_2_	Nitrogen dioxide
SO_2_	Sulfur dioxide
O_3_	Ground-level ozone
PM	Particulate matter
WHO	World Health 232 Organization
LCA	Life cycle assessment
HC	Hydrocarbons
NMVOC	Non-methane volatile organic compounds
Cd	Cadmium
As	Arsenic
Pb	Lead
Hg	Mercury
C_6_H_4_Cl_2_	Dichlorobenzene
VOC	Volatile organic compounds
PAHs	Polycyclic aromatic hydrocarbons
CAGR	Compound annual growth rate
LCC	Life cycle costing
LCCA	Life cycle cost analysis
WEO	Waste engine oil
WVO	Waste vegetable oil
OMNC	Organo-modified nano clay
SiO_2_	Silica
ZnO	Zinc oxide
CaCO_3_	Calcium carbonate
Al_2_O_3_	Aluminum oxide
Fe_2_O_3_	Iron oxide
TiO_2_	Titanium dioxide
CNT	Carbon nano tube
LDPE	Low-density polyethylene
HDPE	High-density polyethylene
AFM	Atomic Force Microscopy
FTIR	Fourier Transform Infrared Spectroscopy
ER	Energy ratio
SFE	Surface free energy
TSR	Tensile strength ratio
ITS	Indirect tensile strength
RAP	Recycled asphalt pavement
WMA	Warm mix asphalt
NA	Natural asphalt
RA	Rock asphalt
TLA	Trinidad lake asphalt
BGA	Buton granular asphalt
ASB	Asbuton
BioCS	Bio-oil of coconut shells
VR	Vegetable resin
PEW	Polyethylene wax
WOO	Waste olive oil
O/R	Oil/resin ratios
REOBs	Re-refined engine oil bottoms
USEPA	United States Environmental Protection Authority
PCMs	Phase change materials
VO_2_	Vanadium dioxide
PEGs	Polyethylene glycol
